# The Accuracy of Maxillary Canines’ Rotation with Different Attachment Designs: A Retrospective Study

**DOI:** 10.3390/jcm15020632

**Published:** 2026-01-13

**Authors:** Edoardo Staderini, Marianna Balacco, Federica Guglielmi, Michele Tepedino, Anna Alessandri-Bonetti, Rosalba Diana, Licia Leccese, Massimo Cordaro, Patrizia Gallenzi

**Affiliations:** 1 Department of Head and Neck and Sense Organs, A. Gemelli University Policlinic IRCCS, Catholic University of Sacred Heart, 00168 Rome, Italy; edoardo.staderini@unicatt.it (E.S.); marianna.balacco01@icatt.it (M.B.); anna.alessandribonetti@unicatt.it (A.A.-B.); massimo.cordaro@unicatt.it (M.C.); patrizia.gallenzi@unicatt.it (P.G.); 2 Postgraduate School of Orthodontics, Catholic University of the Sacred Heart, 00168 Rome, Italy; rosalbadiana@yahoo.it; 3 Postgraduate School of Pediatric Dentistry, Catholic University of the Sacred Heart, 00168 Rome, Italy; 4 Department of Applied Clinical Sciences and Biotechnology, University of L’Aquila, 67100 L’Aquila, Italy; michele.tepedino@univaq.it; 5 School of Dentistry, Catholic University of the Sacred Heart, Largo Agostino Gemelli 8, 00168 Rome, Italy; licia.leccese01@icatt.it

**Keywords:** clear aligners, attachment design, rotational accuracy, maxillary canine, accuracy

## Abstract

**Background/Objectives**: The rotation of maxillary canines represents one of the least predictable movements with clear aligners, particularly in cases requiring rotations greater than 10°, due to the rounded crown morphology and limited aligner grip. The aim of this retrospective study was to compare three different crescent-shaped attachment designs (vertical, horizontal, and oblique) for maxillary canine rotations greater than 10° with clear aligners. **Methods**: Seventy-eight maxillary canines were retrospectively selected and allocated into three equal groups (*n* = 26) according to the orientation of the applied attachment: vertical, horizontal, or oblique crescent-shaped attachments. Digital STL models (initial, predicted, and final) were imported into Dolphin 3D software 12.0.63 to assess the accuracy of maxillary canine’s rotation through the comparison between planned and achieved values. **Results**: Mean rotational accuracy was 55.10% ± 15.60 for the vertical group, 62.40% ± 16.10 for the horizontal group, and 64.60% ± 19.40 for the oblique group. One-way ANOVA showed no statistically significant differences among groups (*p* = 0.09). Pairwise analysis revealed a statistically significant difference between the oblique and vertical designs (*p* = 0.05). **Conclusions**: Attachment orientation may influence the accuracy of maxillary canine rotation with clear aligners, with oblique crescent-shaped attachments showing a trend toward improved rotational control.

## 1. Introduction

Clear aligner therapy has become increasingly popular among adult patients due to its esthetic appeal and improved comfort compared with fixed orthodontic appliances [1]. Despite continuous technological advancements, the predictability of certain orthodontic movements remains limited. Among these, the rotation of teeth with rounded crown morphology, such as maxillary canines, has consistently been reported as one of the most challenging movements to achieve accurately [2,3]. According to a recent meta-analysis by Koletsi et al. 2021 [4], the accuracy of maxillary canines’ rotation with clear aligners—the comparison between software-predicted and clinically achieved outcomes—is 47.90%. From a biomechanical perspective, this limited accuracy can be explained by the geometry of the tooth. Cylindrical or rounded crown morphologies, such as those of maxillary canines, provide shorter lever arms and reduced reactive surfaces for aligner engagement, thereby diminishing the aligner’s ability to generate effective rotational moments and increasing the likelihood of slippage at the aligner–tooth interface [5]. These findings are corroborated by observational studies. Bilello et al. (2022) [6] reported a statistically significant discrepancy between the planned and achieved rotation of maxillary canines, while Lombardo et al. (2017) [7] identified mandibular canine rotation as the least predictable movement (54.20%), indicating a similar trend for upper canines [8]. The limited effectiveness of rotational forces with clear aligners may be due to poor aligner fit on the convex morphology of the canine crown, a condition frequently observed when attachments are not employed [9]. The anatomical shape of maxillary canines, characterized by a convex and nearly cylindrical crown, reduces the available surface area for aligner contact on the mesial and distal aspects. This morphology shortens the biomechanical lever arm and limits the formation of stable active and reactive surfaces, resulting in reduced rotational control, lower accuracy, and a higher tendency toward undesired movements such as intrusion [5,10]. The use of auxiliary elements such as attachments may improve aligner fit and enhance the control of rotational forces [11,12,13]. Among the various attachment designs proposed for rotational control, a recent paper showed that oblique crescent-shaped attachments with different orientations may offer specific biomechanical advantages for tooth rotations [14]. Specifically, the inclination of the active surface—whether vertical, horizontal, or oblique—may influence both the rotational efficacy and the control of intrusive side effects (the so-called “screwdriver effect”). The morphology of attachments has proven to be a key factor in enhancing force transmission and achieving more predictable tooth movements. Sultanoğlu et al. 2024 [15] further investigated the impact of different attachment types and positions on rotational movement in clear aligner treatments. Their finite element analysis revealed that horizontal rectangular attachments placed on both buccal and lingual surfaces resulted in the highest tooth displacement (0.1267 mm) and the greatest stress in the periodontal ligament (0.1971 MPa), indicating enhanced rotational control [16]. Conversely, semi-ellipsoid attachments in similar positions led to the least aligner displacement (0.1441 mm), suggesting improved retention.

However, there is limited clinical evidence concerning the gold-standard attachment’s morphology and size for the rotation of maxillary canines with clear aligners.

The aim of the present study was to compare the accuracy of three different attachment designs for maxillary canines’ rotation with clear aligners. The null hypothesis is that the attachment design (vertically, horizontally, and obliquely oriented crescent-shaped) does not influence the accuracy of canine rotation.

## 2. Materials and Methods

This retrospective study was designed following the STrengthening the Report of OBservational studies in Epidemiology (STROBE) guidelines for observational studies (Supplementary Material S1) [17]. This retrospective controlled study was approved by the Institutional Review Board of Policlinico Agostino Gemelli—CET Lazio Area 3 (Protocol n° 0029107/22 of 14 September 2022). The study is in accordance with the Declaration of Helsinki from 1975 and subsequent revisions and written informed privacy policies were obtained from all the subjects who took part in the study.

### 2.1. Participants

A total of 182 records of patients treated with clear aligners between September 2022 and June 2023 were retrospectively screened. This time interval reflects routine clinical treatments performed using contemporary aligner materials, software, and attachment libraries still currently in use. A follow-up period was necessary to ensure treatment completion and availability of post-treatment STL files. All patients were treated using the Nuvola^®^ system (DSD, Rome, Italy). The aligners were made of Polyethylene Terephthalate Glycol (PET-G), a thermoplastic material characterized by high elasticity and constant force delivery. The standardized treatment protocol followed a specific staging of 0.20 mm of linear displacement and 2.0° of rotation per aligner step.

To ensure the accuracy of the measurements, no virtual overcorrections were programmed in the digital setups (PRED) for the investigated movements. The crescent-shaped attachments (vertical, horizontal, and oblique) were standardized with a thickness of 1.00 mm and a height of 3.00 mm and were positioned at the geometric center of the clinical crown using manufacturer-provided bonding templates. Furthermore, cases requiring interproximal reduction (IPR) on the canines or the use of adjunctive auxiliaries (such as buttons, elastics, or power arms) were excluded from the sample to isolate the biomechanical effect of the attachment orientation alone. The three groups were matched for age and sex to ensure baseline comparability and minimize confounding variables. All the eligible patients were provided with written informed consent forms and privacy policies, accompanied by appropriate verbal explanations. All subjects agreeing to participate were enrolled in the study.

The subjects were retrospectively selected based on the following inclusion criteria:

- Adult patients aged between 18 and 35 years;
- Presence of a permanent complete dentition, excluding third molars;
- Moderate anterior crowding, defined as a PAR index anterior segment score of 2–3 [18];
- Absence of tooth agenesis;
- No history of previous orthodontic treatment.

Exclusion criteria were as follows:

- Presence of dental prostheses, crowns, or restorations involving canines;
- Periodontal disease or bone loss affecting the maxillary arch;
- Systemic conditions affecting bone metabolism (e.g., diabetes, osteoporosis);
- Missing or impacted maxillary canines;
- Use of attachments or auxiliaries not following the standardized protocol;
- Unavailability of digital records or lack of pre- or post-treatment STL scans.
- Use of interproximal reduction (IPR) on the investigated canines or adjunctive procedures (e.g., buttons, elastics, or power arms).

After the application of the inclusion and exclusion criteria, 78 maxillary canines were eligible for further analysis. Each maxillary canine included in the study originated from a different patient, resulting in a total of 78 patients contributing one tooth each. No patient contributed bilateral canines to the dataset. This methodological approach ensured statistical independence across observations, thereby satisfying the assumptions required for subsequent one-way ANOVA testing. Three groups were defined based on the type of composite attachment employed:

- Vertical crescent-shaped attachments (vertical group);
- Horizontal crescent-shaped attachments (horizontal group);
- Oblique crescent-shaped attachments (oblique group).

Each group included 26 maxillary canines. The sample size was determined through a power analysis conducted using G*Power software (version 3.1.9.7). Assuming a medium effect size (f = 0.40), an alpha level of 0.05, and a power of 0.80 for a one-way ANOVA with three groups, a total sample of 66 canines was required. To further increase the robustness of the study and account for potential outliers, the sample was expanded to include 78 canines. To ensure a balanced design for statistical comparisons, a target of 26 eligible canines per group was set; recruitment for each group was concluded once this predetermined threshold was reached from the available retrospective pool [19].

**Figure 1:**
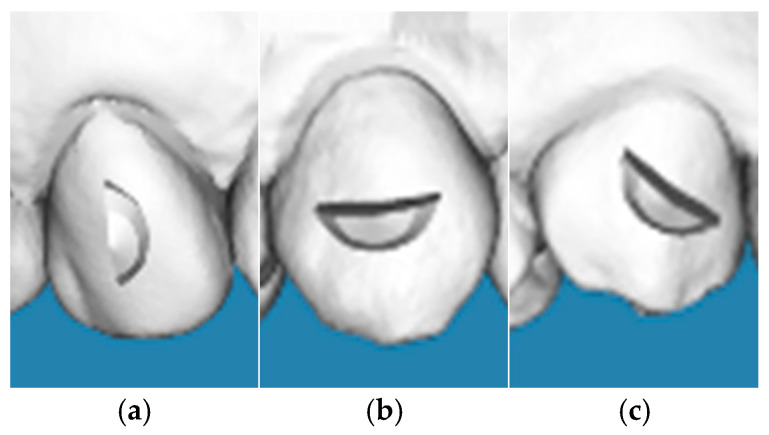
Schematic illustration of the crescent-shaped attachment designs and their orientations on the maxillary canine: (**a**) vertical orientation, (**b**) horizontal orientation, and (**c**) oblique orientation. All attachments feature standardized dimensions (3.00 mm height and 1.00 mm thickness) and are positioned at the geometric center of the clinical crown.

All cases were treated with approximately 23 ± 3 aligners, following a standardized protocol in which the only variable was the attachment design. The attachment positioning and shape were consistent within each group to minimize biomechanical variability. Each attachment was placed at the geometric center of the clinical crown, standardized in both vertical and mesiodistal dimensions, to ensure consistency in positioning among all specimens. Although the study design was retrospective, attachment standardization was ensured by including only cases treated according to a predefined institutional protocol. All attachments were digitally planned through the same aligner software and attachment library. Attachment dimensions (morphology, height and thickness) were standardized across all groups; the attachments’ orientation (vertical, horizontal, or oblique) represented the only experimental variable (Figure 1). Clinically, attachments were bonded using manufacturer-provided templates. Cases not adhering to this protocol were excluded from the analysis.

### 2.2. Measurements

For each patient, three digital models in STL format were obtained: the pre-treatment model (PRE), representing the initial malocclusion; the predicted model (PRED), corresponding to the virtual simulation of the planned tooth movement with clear aligners; and the post-treatment model (POST), reflecting the final occlusal outcome after treatment completion.

All STL files were imported into Dolphin 3D software 12.0.63 (Dolphin Imaging & Management Solutions, Chatsworth, CA, USA) and aligned using the software’s internal reference coordinate system. Calibration for linear and angular measurements was performed through standardized model orientation prior to analysis, ensuring consistency across all measurements.

**Figure 2:**
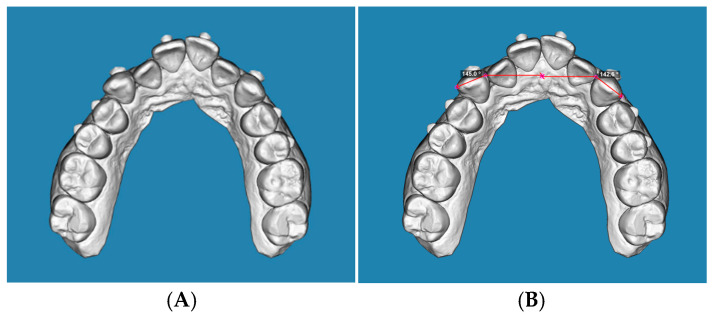
(**A**): Occlusal view of an initial STL digital model, (**B**): occlusal view of an initial STL digital model, displaying the angular measurement used to assess the maxillary canine rotation accuracy, based on the angle between the mesio-distal contact line and the perpendicular to the mid-palatal raphe.

**Figure 3:**
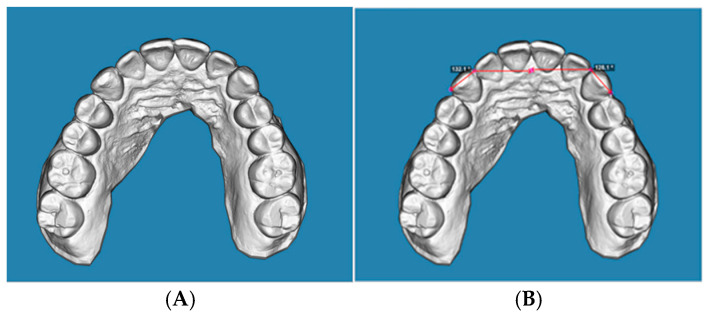
(**A**): Occlusal view of a predicted STL digital model, (**B**): occlusal view of a predicted STL digital model, displaying angular measurements.

**Figure 4:**
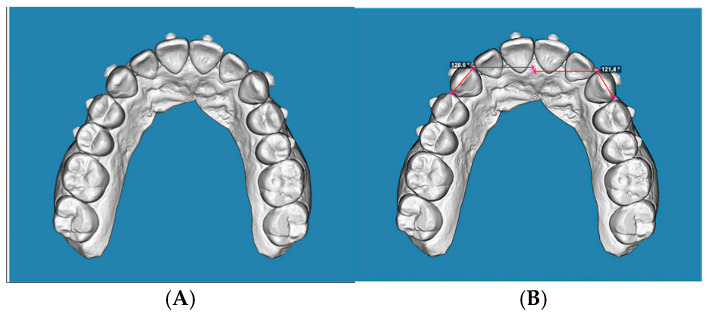
(**A**): Occlusal view of a final STL digital model, (**B**): occlusal view of a final STL digital model, displaying angular measurements.

Measurements were performed on the occlusal view. According to the protocol proposed by Nahidh et al. [17], rotation was defined as the angle between a line connecting the mesial and distal contact points of the canine and a line drawn perpendicular to the mid-palatal raphe, passing through the mesial contact point (Figure 2A,B, Figure 3A,B and Figure 4A,B). The selected reference points were cross-checked from the sagittal view to confirm their three-dimensional accuracy and consistency across the initial, predicted, and final models.

The same angular measurement described above was systematically performed on each of the three STL models—initial (PRE), predicted (PRED), and final (POST). In each case, the angle formed by the mesial-distal contact line of the maxillary canine and the perpendicular to the mid-palatal raphe was measured consistently across all timepoints.

The maxillary canine rotation accuracy was calculated by comparing the predicted (PRED) and the achieved (POST) rotation values.

The percentage of rotation accuracy was calculated according to the formula originally described by Kravitz et al. (2008) [19]:

$$\mathrm{Accuracy}\;(\%)\;=\;\left[\left(\frac{Achieved\;Rotation}{Predicted\;Rotation}\right)\times 100\right]$$

To account for the discrepancy between planned and achieved movements, the following formula based on the relative error was applied

$$\mathrm{Accuracy}\;(\%)=100\%-\left[\left(\frac{I\;predicted-achieved\;I}{I\;predicted\;I}\right)\times 100\%\right]$$

All measurements were independently performed by two experienced examiners (MB and RD) who were fully blinded to the attachment group assignment during the entire assessment process. To ensure standardized conditions, all digital analyses were conducted using the same computer workstation in a quiet environment with consistent lighting. To minimize measurement errors, all 78 models were re-analyzed by both operators after a fortnightly interval under identical conditions. Intra- and inter-operator reliability were assessed using intraclass correlation coefficients (ICC) based on a two-way random-effects model with absolute agreement. All ICC values demonstrated excellent reliability (ICC ≥ 0.93), as detailed in Table 1.

### 2.3. Statistical Analysis

The sample size of this study was estimated according to the formula proposed by Wang et al. (2016) [20] for comparing group means:

N=2σ2(z1−β+z1−α2)(μ0−μ1)2

where *N* is the required sample size per group, σ is the standard deviation of the study population, z is the critical value of the standard normal distribution for a given α or β, α is the probability of Type I error, β is the probability of Type II error, μ_0_ is the population mean, and μ_1_ is the expected mean of the study group. The term Δ = (μ_1_ − μ_0_) represents the expected mean difference between groups, that is, the minimum difference in rotational accuracy considered relevant to detect.

Assuming a Type I error rate of 0.05 and a statistical power (1 − β) of 0.80, the required sample size was estimated a priori based on previously published data on rotational accuracy with aligners. Kravitz et al. (2009) [21] reported a mean accuracy of 32.20% with a standard deviation of 28.60% (*n* = 57) for maxillary canine rotation. This value was adopted to represent the expected within-group variability (σ) in the present investigation. The expected mean difference (Δ) was set at 18 percentage points, reflecting a moderate and clinically meaningful variation in rotational accuracy between the most and least effective attachment designs (e.g., oblique vs. vertical). The choice of a medium effect size (f = 0.40) was considered appropriate to balance clinical relevance with the high inherent variability of clear aligner outcomes reported in the literature. This parameter ensures the study is powered to detect the aforementioned difference, which represents a clinically meaningful threshold for orthodontic decision-making [19]. Based on these parameters (σ = 28.60%, Δ = 18%, α = 0.05, power = 0.80), the formula proposed by Wang et al. (2016) [20] yields approximately 26 teeth per group, corresponding to 78 teeth in total for a three-group comparison. The current study therefore meets the theoretical sample size required to detect such a difference with 80% statistical power, supporting the adequacy and feasibility of the study design relative to previously published work (Kravitz et al., 2009) [21].

Descriptive statistics were calculated for all variables. Intra- and inter-operator reliability for all measurements (PRE, POST, and PRED values) were assessed through the calculation of the intraclass correlation coefficient (ICC) based on a two-way random-effects model with an agreement definition. Accordingly, the average of the two operators’ measurements was used for each case in subsequent analyses. Absolute values were considered to underline the accuracy of the movement without the influence of the direction of the movement (clockwise vs. counterclockwise rotation).

The Shapiro–Wilk test was applied to assess the normality of the distribution of values within each group. Based on the results of the normality tests, appropriate statistical comparisons were selected. When the assumption of normality was not satisfied, Kruskal–Wallis non-parametric tests was used to evaluate differences in the prescribed rotational movement among the three groups (vertical, horizontal, and oblique). The same non-parametric approach was used to compare rotational accuracy across groups. Post hoc comparisons were carried out using Dunn’s test with Bonferroni-adjusted *p*-values. A significance level of 0.05 was set for all tests. To account for multiple comparisons and minimize the risk of Type I error, a Bonferroni correction was applied to all pairwise comparisons. Additionally, to assess the clinical magnitude of the differences beyond *p*-values, effect sizes (Cohen’s d) were calculated. Effect sizes were interpreted as small (0.20), medium (0.50), or large (0.80). Statistical analysis was performed using R software 4.4.3 (2025-02-28) (R Foundation for Statistical Computing, Vienna, Austria).

## 3. Results

All ICC values indicated excellent intra- and inter- operator reliability (Table 1). Intraclass correlation coefficients were computed using all angular measurements (PRE, PRED, and POST) performed by operator 1 and operator 2, with each operator repeating the measurements twice. For graphical illustration purposes, intra- and inter-operator agreement was visualized using pre-treatment angular measurements as representative examples of measurement repeatability.

Descriptive statistics for rotational accuracy across the three attachment groups are reported in Table 2. The vertical crescent-shaped attachment group exhibited the lowest mean accuracy (55.05%, sd = 15.60), with a minimum value of 22.20% and a maximum of 89.01%. The horizontal group showed a higher mean accuracy of 62.44% (sd = 16.07). The highest mean accuracy was observed in the oblique crescent-shaped attachment group (64.64%, sd = 19.40).

**Figure 5:**
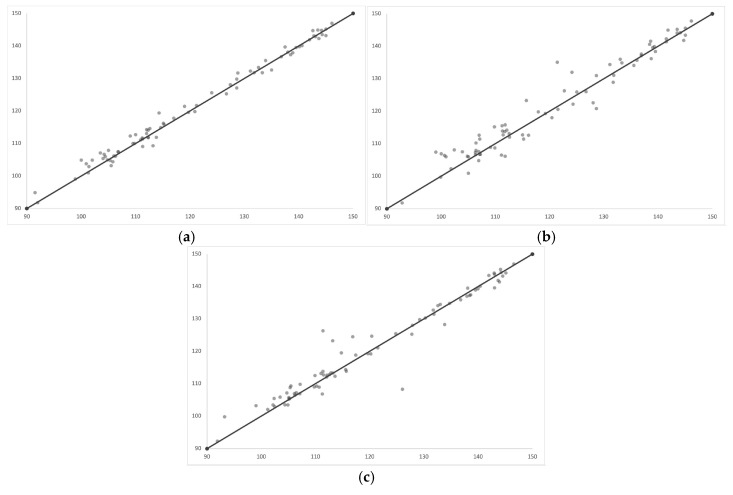
(**a**) Intra-operator agreement for operator 1 between repeated pre-treatment (PRE) angular measurements; (**b**) Intra-operator agreement for operator 2 between repeated pre-treatment (PRE) angular measurements; (**c**) Inter-operator agreement between operator 1 and operator 2 based on pre-treatment (PRE) angular measurements.

The Shapiro–Wilk test was performed to assess the normality of the distribution of accuracy values within each attachment group. In all groups, the results indicated no significant deviation from normality (*p* > 0.05) (Table 1) (Figure 5).

**Figure 6:**
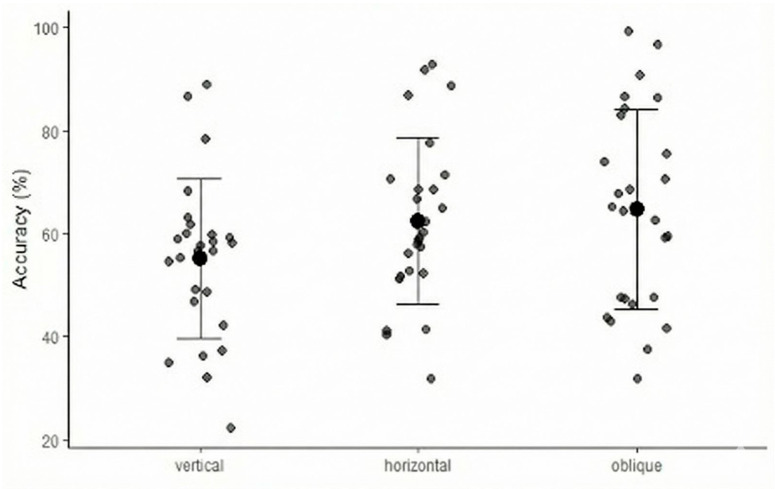
Scatter plot of rotational accuracy values across the three attachment groups.

The results of the one-way analysis of variance (ANOVA) revealed no statistically significant difference (*p* = 0.09) in rotational accuracy among the three attachment groups (vertical group, horizontal group, oblique group) (Table 2) (Figure 6).

As illustrated in Figure 6, the distribution of individual accuracy values highlights a high degree of inter-individual variability in the expression of rotational movement within each cohort. Specifically, the wide range between minimum and maximum accuracy values—ranging from 22.20% in the vertical group to 99.40% in the oblique group—indicates that while the mean accuracy improved with oblique designs, individual responses varied significantly. This substantial within-group variance, clearly visible from the dispersion of data points, is the primary factor contributing to the lack of overall statistical significance in the ANOVA (*p* = 0.09), despite the observable trend favoring the oblique orientation.

To explore specific group-level differences in rotational accuracy, pairwise comparisons between the attachment groups were conducted using independent-samples *t*-tests.

The comparison between the vertical and horizontal groups did not reveal a statistically significant difference in rotational accuracy (*p* = 0.06), although a trend toward higher accuracy was observed in the horizontal group. A statistically significant difference was found between the vertical and oblique attachment groups (*p* = 0.05), with the oblique group demonstrating greater rotational accuracy. No significant difference was observed between the horizontal and oblique groups (*p* = 0.74). Pairwise *t*-tests results are reported in table (Table 3). The pairwise comparison between the vertical and oblique groups yielded a borderline significant *p*-value (*p* = 0.05) accompanied by a moderate effect size (Cohen’s d = 0.54). This indicates that while the overall ANOVA (*p* = 0.09) suggests a shared variance among groups, the specific difference between the vertical and oblique designs represents a clinically relevant trend.

## 4. Discussion

The present study investigated the influence of crescent-shaped attachment orientation on the rotational accuracy of maxillary canines treated with clear aligners.

Baseline comparability among the groups confirmed the homogeneity of the sample. The mean accuracy of maxillary canines’ rotation was 60.70% indicating overall clinical effectiveness across all designs. While the overall ANOVA did not reach statistical significance (*p* = 0.09) among the three attachment groups, pairwise analysis revealed a statistically significant difference between the oblique attachments and the vertical design (*p* = 0.05), favoring the former. From a clinical standpoint, the observed difference between the vertical (55.05%) and oblique (64.64%) attachment groups suggests that the orientation of the active surface plays a role in the effectiveness of force transmission.

Based on these findings, the null hypothesis—stating that attachment orientation does not affect the rotational accuracy—cannot be fully rejected in the overall model, the findings should be interpreted as indicative of a trend toward improved rotational control with oblique crescent-shaped attachments rather than definitive superiority, given the borderline significance (*p* = 0.05) and the moderate effect size (d = 0.5) observed.

Although the study results suggest that the inclination of the active surface may play a clinically relevant role in enhancing rotational accuracy, the fact that even the best-performing group remained below the 65% threshold implies that clinicians should still anticipate a ‘tracking loss’ during complex canine rotations. Practically, choosing an oblique orientation may result in a final tooth position that is closer to the digital plan, thereby potentially reducing the number of aligners required during the refinement phase, even if it does not eliminate the need for refinement entirely. Despite the trends toward improved control with oblique designs, it is noteworthy that all attachment configurations demonstrated rotational accuracy values below 65%. This confirms that canine rotations exceeding 10° remain a biomechanically demanding movement for clear aligners, often necessitating refinements [22,23].

The wide scatter distribution of individual values and the broad range of accuracy outcomes highlight the clinical unpredictability inherent in clear aligner therapy for complex movements. This suggests that while attachment geometry is a critical factor in optimizing force transmission, it is not the sole determinant of success. Factors such as individual crown morphology, aligner seating, and the biological response of the periodontal ligament likely introduce the ‘noise’ observed in the data. Consequently, clinicians should interpret the trend toward higher accuracy with oblique attachments as a way to improve the probability of success, while acknowledging that a significant proportion of cases will still require refinements or specific overcorrection protocols due to high individual variability. The findings of the present investigation demonstrate a notably higher rotational accuracy for maxillary canines compared to earlier clinical studies. Specifically, Kravitz et al. (2009) [21] reported a mean accuracy of only 32.20% for canine rotation, whereas our results indicate an overall clinical effectiveness of approximately 60.70% across all attachment designs. This significant improvement—nearly double the accuracy reported a decade ago—can likely be attributed to the evolution of aligner materials and more sophisticated digital planning algorithms. Contemporary thermoplastic polymers provide more constant force delivery compared to earlier materials, which may explain the enhanced expression of planned movements [24]. Furthermore, our data show higher predictability than the 47.90% accuracy estimated in the meta-analysis by Koletsi et al. (2021) [4]. While that meta-analysis identified canine rotation as one of the least predictable movements, our results suggest that using specific crescent-shaped geometries can push this accuracy above 64.00%. This supports the findings of Cortona et al. (2020) [1], who emphasized that even a single attachment significantly improves the control of teeth with rounded anatomies by providing a stable reactive surface [24].

Regarding the study period, data were collected between September 2022 and June 2023. This timeframe remains highly relevant to current technology, as it reflects the use of contemporary aligner materials, digital software, and attachment libraries that are still the gold standard in clinical practice. Furthermore, this retrospective window was necessary to ensure the completion of treatments and the availability of post-treatment STL files required for accuracy assessment.

From a methodological standpoint, each maxillary canine included in the dataset originated from a different patient. By ensuring that no patient contributed bilateral canines, the study avoided potential clustering effects and satisfied the assumptions of statistical independence required for the one-way ANOVA.

The scatter distribution of individual accuracy values (Figure 6) highlights a wide inter-individual variability in maxillary canine rotation achieved with clear aligners, regardless of attachment orientation. Although oblique attachments demonstrated higher mean accuracy values compared with vertical and horizontal designs, substantial overlap among groups was observed, with accuracy ranging from low to high values in all attachment configurations. This finding suggests that while attachment orientation may influence rotational efficiency on average, it does not ensure predictable clinical outcomes at the individual level. The presence of both high and low responders within each group indicates that additional factors—such as initial severity of rotation, crown morphology, aligner fit, and patient compliance—likely play a relevant role in determining treatment accuracy. From a clinical perspective, oblique attachments may therefore increase the probability of achieving higher rotational accuracy but should not be considered a guarantee of successful maxillary canine rotation with clear aligners, emphasizing the need for careful case selection and, when appropriate, adjunctive strategies or alternative mechanics.

From a biomechanical perspective, the superiority of the oblique crescent-shaped attachment observed in our study (*p* = 0.05 vs. vertical) aligns with recent finite element analysis (FEA) research. Sultanoğlu et al. (2024) [15] demonstrated that attachment orientation and position are critical for maximizing tooth displacement and managing stress distribution in the periodontal ligament [25]. Our study clinically corroborates the hypothesis that an inclined active surface generates a more effective rotational moment while simultaneously mitigating the ‘screwdriver effect’ (unwanted intrusion), a common side effect of rotational forces on convex crowns [26]. This finding suggests that clinicians should move beyond standard vertical attachments in favor of more customized, biomechanically driven designs for complex rotations.

The rounded crown morphology of the maxillary canine limits the aligner’s ability to establish a stable grip, leading to a loss of tracking as the rotational movement progresses. Consequently, while the use of oblique crescent-shaped attachments may enhance treatment efficiency by achieving a outcome closer to the digital plan, clinicians should still expect the need for refinements in most cases involving complex rotations. The value of selecting an optimized attachment design lies in reducing the extent of these refinements and potentially decreasing the total number of additional aligners required to reach the final goal, thereby improving overall practice workflow and patient satisfaction.

When evaluating these results against a clinical ‘gold standard’, such as traditional fixed appliances, a mean accuracy of 60.70% might be interpreted as a suboptimal outcome. While this represents an improvement over earlier aligner studies, it highlights a fundamental limitation of clear aligner biomechanics in managing complex canine rotations. In many clinical scenarios, an accuracy threshold of approximately 60% implies a significant discrepancy between the digital plan and the final clinical position, which essentially constitutes a ‘tracking failure’. Therefore, clinicians should be aware that for rotations exceeding 10°, clear aligners frequently fail to achieve complete movement expression. In these cases, the use of optimized oblique attachments is not a guarantee of total success, but rather a strategy to mitigate error. If near-perfect rotational control is required, clinicians should consider adjunctive mechanics or acknowledge that aligners alone may be insufficient for the task.

### 4.1. Strengths and Limitations

To the best of our knowledge, this is the first clinical investigation to analyze how different orientations of crescent-shaped attachments influence the rotational accuracy of maxillary canines. The results provide a foundation for developing a biomechanically driven strategy to optimize attachment design and enhance treatment accuracy. Another methodological strength is the assessment of measurement reliability: rotational measurements were performed independently by two operators, and inter-rater reliability was verified through intra-class correlation coefficients (ICCs) (Table 1) demonstrating a high level of agreement and thus strengthening the validity of the results.

The main limitation was the retrospective design; however, the strict inclusion criteria improved population homogeneity and minimized selection bias.

A control group treated without attachments was not included; nonetheless, this limitation is balanced by strong clinical evidence showing that the investigated type of movement is less predictable without attachments [25].

Measurement accuracy may be affected by operator-dependent variability in landmark identification. This issue was minimized by using a standardized measurement protocol; moreover, independent measurements were performed by two experienced operators, with excellent intra- and inter-operator reliability (ICC ≥ 0.93).

Additionally, the study did not evaluate the presence or morphology of attachments on adjacent teeth because of variability in the clinical records. The main effort was to standardize the canine attachment design, because the study specifically focused on evaluating the impact of its orientation.

Despite these limitations, which may affect generalizability, the internal validity of the study remains robust.

### 4.2. Future Research Perspectives

The findings of the present study should be considered to be preliminary but informative, providing a basis for future prospective studies with larger and more diverse samples.

Future research should specifically examine the role of attachments placed on adjacent teeth, as their morphology and positioning may significantly influence rotational accuracy by modifying aligner fit and force distribution. In addition, the interaction between canine rotation and other biomechanical movements, such as intrusion or vertical control, deserves closer evaluation. These concomitant movements may alter the vector of applied forces, thereby affecting the accuracy of rotational outcomes. A more comprehensive understanding of these biomechanical interactions would contribute to the development of optimized protocols and would improve the clinical effectiveness of clear aligner therapy.

## 5. Conclusions

The present retrospective study evaluated the impact of different crescent-shaped attachment orientations on the rotational accuracy of maxillary canines. Based on the findings, the following conclusions can be drawn:

- Attachment Orientation: Attachment orientation may influence the rotational accuracy of maxillary canines. Oblique crescent-shaped attachments demonstrated a clinical trend toward improved rotational control compared with vertical designs (*p* = 0.05; Cohen’s d = 0.54), likely due to more effective rotational force transmission.
- Rotational Accuracy Thresholds: The oblique crescent-shaped attachment achieved the highest mean accuracy at 64.64% ± 19.40%. However, all tested configurations showed mean accuracy values below 65%, confirming that maxillary canine rotations greater than 10° remain a biomechanically challenging movement.
- Clinical Predictability and Variability: While specialized attachment designs optimize force transmission, significant inter-individual variability still remains. This variability suggests that biological factors and individual crown morphology play a substantial role in treatment outcomes.
- Methodological Robustness: By including only one canine per patient, this study ensured statistical independence and avoided clustering effects, providing a reliable baseline for the clinical effectiveness of these contemporary attachment designs.
- Clinical Recommendation: For complex canine rotations (>10°), clinicians should consider the use of oblique crescent-shaped attachments to enhance predictability, while remaining aware that refinements or overcorrection protocols are frequently necessary to achieve planned outcomes.
- Future Research: Further prospective clinical trials with larger sample sizes are required to investigate the interaction between canine attachments and those on adjacent teeth, as well as the impact of different thermoplastic materials on these specific force systems.

## 6. Highlights

- The present study showed that attachment geometry could optimize maxillary canine’s rotation, particularly for complex movements (>10°).
- The highest accuracy was observed in the oblique crescent-shaped attachment group (mean: 64.64%, σ = 19.40), with values ranging from 31.74% to 99.40%.

## Figures and Tables

**Table 1:** Intra-operator and Inter-operator reliability (Intraclass Correlation Coefficient). ICC(C,1) = intraclass correlation coefficient, two-way mixed-effects model (consistency); ICC(A,1) = intraclass correlation coefficient, two-way random-effects model (absolute agreement); 95% CI = 95% confidence interval.

Comparison Type	ICC Model	ICC Value	95% CI
Intra-operator 1	ICC(C,1)	0.98	0.97–0.98
Intra-operator 2	ICC(C,1)	0.93	0.91–0.94
Inter-operator 1–2	ICC(A,1)	0.93	0.91–0.94

**Table 2:** Descriptive statistics of rotational accuracy.

Attachment Group	*n*	Mean Accuracy (%)	SD	Min (%)	Max (%)
Vertical crescent-shaped	26	55.05	15.60	22.20	89.01
Horizontal crescent-shaped	26	62.44	16.07	31.66	92.98
Oblique crescent-shaped	26	64.64	19.40	31.74	99.40

**Table 3:** Pairwise comparisons between attachment groups (*t*-tests). * Statistically significant for *p* < 0.05.

Attachment Group	t-Value	*p*-Value
Vertical crescent-shaped	−1.89	0.06
Horizontal crescent-shaped	−2.04	0.05 *
Oblique crescent-shaped	−0.34	0.74

## Data Availability

The datasets generated and/or analyzed during the current study are available from the corresponding author on reasonable request. The data are not publicity available due to privacy issues.

## Supplementary Materials

The following supporting information can be downloaded at: https://www.mdpi.com/article/10.3390/jcm15020632/s1, File S1: STROBE Statement—Checklist of items that should be included in reports of cohort studies.

## Abbreviations

The following abbreviations are used in this manuscript:

PRE	pre-treatment model
PRED	predicted model
POST	post-treatment model
ICC	intraclass correlation coefficient

## References

[1] Cortona A., Rossini G., Parrini S., Deregibus A., Castroflorio T. (2020). Clear aligner orthodontic therapy of rotated mandibular round-shaped teeth: A finite element study. Angle Orthod..

[2] Haouili N., Kravitz N.D., Vaid N.R., Ferguson D.J., Makki L. (2020). Has Invisalign improved? A prospective follow-up study on the efficacy of tooth movement with Invisalign. Am. J. Orthod. Dentofac. Orthop..

[3] Charalampakis O., Iliadi A., Ueno H., Oliver D.R., Kim K.B. (2018). Accuracy of clear aligners: A retrospective study of patients who needed refinement. Am. J. Orthod. Dentofac. Orthop..

[4] Koletsi D., Iliadi A., Eliades T. (2021). Predictability of rotational tooth movement with orthodontic aligners comparing software-based and achieved data: A systematic review and meta-analysis of observational studies. J. Orthod..

[5] Aminian A., Garino F., Castroflorio T., Younessian F. (2024). Biomechanics of tooth rotation in clear aligner therapy. Semin. Orthod..

[6] Bilello G., Fazio M., Amato E., Crivello L., Galvano A., Currò G. (2022). Accuracy evaluation of orthodontic movements with aligners: A prospective observational study. Prog. Orthod..

[7] Lombardo L., Arreghini A., Ramina F., Ghislanzoni L.T.H., Siciliani G. (2017). Predictability of orthodontic movement with orthodontic aligners: A retrospective study. Prog. Orthod..

[8] Manni A., Migliorati M., Boggio A., Drago S., Paggi E., Calzolari C., Gastaldi G., Cozzani M. (2024). Evaluation of the Co-Go-Me angle as a predictor in Class II patients treated with Herbst appliance and skeletal anchorage: A retrospective cohort study. Front. Oral Health.

[9] Manni A., Boggio A., Gastaldi G., Cozzani M. (2024). Is significant mandibular advancement possible after the peak of puberty? Dento-osseous palatal expansion and the STM4 technique (Skeletal Therapy Manni Telescopic Herbst 4 miniscrews): A case report. Int. Orthod..

[10] Elkholy F., Mikhaiel B., Repky S., Schmidt F., Lapatki B.G. (2019). Effect of different attachment geometries on the mechanical load exerted by PET-G aligners during derotation of mandibular canines: An in vitro study. J. Orofac. Orthop..

[11] Momtaz P. (2016). The Effect of Attachment Placement and Location on Rotational Control of Conical Teeth Using Clear Aligner Therapy. Master’s Thesis.

[12] Gomez J.P., Peña F.M., Martínez V., Giraldo D.C., Cardona C.I. (2015). Initial force systems during bodily tooth movement with plastic aligners and composite attachments: A three-dimensional finite element analysis. Angle Orthod..

[13] Dasy H., Dasy A., Asatrian G., Rózsa N., Lee H.-F., Kwak J.H. (2015). Effects of variable attachment shapes and aligner material on aligner retention. Angle Orthod..

[14] Hassanaly T., Rabal-Solans A., Mediero-Pérez M.-C., Nieto-Sánchez I. (2024). A comparison of the upper anterior teeth movements with optimized and conventional attachment. J. Clin. Exp. Dent..

[15] Sultanoğlu E., Gürel H.G., Gülyurt M. (2024). The Effects of Different Attachment Types and Positions on Rotation Movement in Clear Aligner Treatments: A Finite Element Analysis. Cureus.

[16] Wen S., Lin D., Yuan X., Wang S., Yang Y., Hu G., Lai W., Long H. (2025). Predictability of tooth derotation with clear aligners and its influencing factors: A retrospective study. Eur. J. Orthod..

[17] Nahidh M., Yassir Y.A. (2023). Methods of measuring distal canine movement and rotation—A review. J. Orthod. Sci..

[18] Richmond S., Shaw W.C., O’Brien K.D., Buchanan I.B., Jones R., Stephens C.D., Roberts C.T., Andrews M. (1992). The development of the PAR Index (Peer Assessment Rating): Reliability and validity. Eur. J. Orthod..

[19] Kravitz N.D., Kusnoto B., Agran B., Viana G. (2008). Influence of attachments and interproximal reduction on the accuracy of canine rotation with Invisalign. A prospective clinical study. Angle Orthod..

[20] Wang M., Spiegelman D., Kuchiba A., Lochhead P., Kim S., Chan A.T., Poole E.M., Tamimi R., Tworoger S.S., Giovannucci E. (2016). Statistical methods for studying disease subtype heterogeneity. Stat. Med..

[21] Kravitz N.D., Kusnoto B., BeGole E., Obrez A., Agran B. (2009). How well does Invisalign work? A prospective clinical study evaluating the efficacy of tooth movement with Invisalign. Am. J. Orthod. Dentofac. Orthop..

[22] Cucchi A., Maiani F., Franceschi D., Sassano M., Fiorino A., Urban I.A., Corinaldesi G. (2024). The influence of vertical ridge augmentation techniques on peri-implant bone loss: A systematic review and meta-analysis. Clin. Implant. Dent. Relat. Res..

[23] Geramy A., Safari F. (2024). Effect of clear aligner type on maxillary full-arch intrusion: 3D analysis using finite element method. BMC Oral Health.

[24] Pede K., Shetty P., Ranjan A., Khan W., Patil H., Mishra H. (2024). Evaluation of effects of different sizes and shapes of attachments during rotation, tipping, and torquing in clear aligner therapy—A finite element study. J. Orthod. Sci..

[25] Jedliński M., Mazur M., Greco M., Belfus J., Grocholewicz K., Janiszewska-Olszowska J. (2023). Attachments for the Orthodontic Aligner Treatment-State of the Art-A Comprehensive Systematic Review. Int. J. Environ. Res. Public Health.

[26] Nucera R., Dolci C., Bellocchio A.M., Costa S., Barbera S., Rustico L., Farronato M., Militi A., Portelli M. (2022). Effects of Composite Attachments on Orthodontic Clear Aligners Therapy: A Systematic Review. Materials.

