# Supplementary material for: The Accuracy of Maxillary Canines’ Rotation with Different Attachment Designs: A Retrospective Study
Source: J Clin Med. 2026 Jan 13;15(2):632. doi: 10.3390/jcm15020632 (PMC12842249; doi:10.3390/jcm15020632)
Supplement: Supplementary file 1 [file jcm-15-00632-s001.zip › jcm-4057265-supplementary.pdf]

STROBE Statement—Checklist of items that should be included in reports of ***cohort studies***

	Item No	Recommendation
**Title and abstract**	1	(*a*) Yes – the study design (“retrospective study”) is stated in the title and abstract, pag. 1
		(*b*) Yes – the abstract provides a balanced summary of objectives, methods, results, and conclusions, pag. 1
**Introduction**		
Background/rationale	2	Yes – the introduction explains the biomechanical limitations of rotations with clear aligners and the rationale for attachment design comparison, pag. 2
Objectives	3	Yes – the objective and null hypothesis are explicitly stated, pag. 2
**Methods**		
Study design	4	Yes – key elements of the retrospective design are stated early, pag. 3
Setting	5	Yes – setting and source of records described; study period partially described, pag. 3
Participants	6	(*a*) Yes – eligibility criteria, selection process, and number of included teeth (n=78 canines) are specified, pag. 3-4
		(*b*) Not applicable – no matched design
Variables	7	Yes – outcomes, exposures, and measurement definitions are clearly provided, pag. 4
Data sources/ measurement	8*	Yes – STL models, Dolphin 3D software, and measurement methods described; comparability between groups ensured, pag. 4
Bias	9	Yes – sources of bias addressed (retrospective design, operator variability, standardization), pag. 9-10
Study size	10	Yes – sample size calculation using Rosner formula is detailed, pag. 6
Quantitative variables	11	Yes – variables treated as continuous; no categorization; formula for accuracy explained, pag. 4-5
Statistical methods	12	(*a*) Yes – ICC, ANOVA, Shapiro–Wilk, t-tests described, pag. 6
		(*b*) Yes – analysis among three attachment groups described, pag. 6
		(*c*) Yes – missing data addressed by exclusion; none present, pag.6
		(*d*) Not applicable – retrospective study based on complete records
		(*e*) No sensitivity analyses conducted
**Results**		
Participants	13*	(a) Yes – numbers at each stage reported: 182 records → 78 included, pag. 3
		(b) Yes – reasons for exclusions correspond to exclusion criteria, pag. 3
		(c) Not included
Descriptive data	14*	(a) Yes – demographic and clinical inclusion characteristics summarized, pag. 3
		(b) Yes – explicitly stated that no missing data were present, pag. 6
		(c) Not applicable (completed treatments only)
Outcome data	15*	Yes – accuracy data reported for all three groups (mean, SD, min, max), pag. 6-7
Main results	16	(*a*) Yes – unadjusted estimates, statistical tests, and 95% CI for ICC reported, pag. 6-7
		(*b*) Not applicable – no categorization
		(*c*) Not applicable
Other analyses	17	Yes – pairwise t-tests between groups
**Discussion**		
Key results	18	Yes – summarized clearly and related to original aims, pag. 8-9
Limitations	19	Yes – limitations described, including retrospective design and absence of no-

		attachment control, pag. 9-10
Interpretation	20	Yes – interpretation cautious and compared with literature, pag. 8-9
Generalisability	21	Yes – external validity briefly discussed (sample and setting), pag. 9
**Other information**		
Funding	22	Yes – “no external funding” clearly stated, pag. 11

*Give information separately for exposed and unexposed groups.

Note: An Explanation and Elaboration article discusses each checklist item and gives methodological background and published examples of transparent reporting. The STROBE checklist is best used in conjunction with this article (freely available on the Web sites of PLoS Medicine at http://www.plosmedicine.org/, Annals of Internal Medicine at http://www.annals.org/, and Epidemiology at http://www.epidem.com/). Information on the STROBE Initiative is available at http://www.strobe-statement.org.
